# Maternal Knowledge, Attitude and Practices toward Free Sugar and the Associations with Free Sugar Intake in Children

**DOI:** 10.3390/nu13124403

**Published:** 2021-12-09

**Authors:** Walaa Abdullah Mumena

**Affiliations:** Clinical Nutrition Department, College of Applied Medical Sciences, Taibah University, P.O. Box 344, Madinah 42353, Saudi Arabia; wmumena@taibahu.edu.sa

**Keywords:** maternal, knowledge, attitude, practices, children, free sugar intake, Saudi Arabia

## Abstract

Research addressing factors related to free sugar (FS) consumption among children in Saudi Arabia is lacking. We aimed to evaluate maternal knowledge, attitude, and practices toward FS and the associations with children’s intake of FS. This cross-sectional study included 424 Saudi children aged 6–12 years and their mothers. Data related to maternal knowledge, attitude, and practices were collected using an online survey. Data concerning children’s habitual intake of FS were collected through phone interviews using a validated food frequency questionnaire. Limited knowledge on FS was observed among mothers of children [median 7.00 [interquartile range 6.00–8.00] out of 11.0. Maternal knowledge was not correlated with maternal attitude or practices toward FS. Maternal knowledge towards FS did not predict children’s intake of FS, whereas maternal attitude and practices toward limiting the consumption of FS predicted lower intake of FS among Saudi children, particularly the FS consumed from solid food sources (B: −5.73 [95% confidence interval (CI): −9.79 to −1.66]) and (B: −6.85 [95% CI: −11.9 to −1.80]), respectively. Despite the limited knowledge pertaining to FS among mothers in Saudi Arabia, they were making efforts to limit their children’s consumption of FS.

## 1. Introduction

Excessive intake of free sugar (FS) among children has been reported in several settings [1,2,3]. High intake of FS has been of concern due to its negative impact on health [4,5]. Existing evidence suggests that excessive intake of sugary food replaces the consumption of important foods in the diet, such as fruits and vegetables, leading to lower intake of crucial nutrients and lower quality of diet [6,7,8]. Insufficient nutrient intake among children for a prolonged period of time may result in growth impairment in addition to many other long-term health consequences [9].

The theory of knowledge, attitude, and practice is important to explain health-related behaviors. The theory consists of three components: acquiring knowledge, generating a belief, and forming a behavior [10]. However, the palatability and convenience of sugary foods could also influence practices related to the consumption of FS foods [11,12,13,14]. In addition, the cost per calorie for energy-dense nutrient-poor foods is generally less, which may influence food-related decisions among individuals and result in excessive intake of FS [15,16].

National interventions have been implemented to reduce the consumption of FS among Saudis, e.g., sugary drink tax policy and food labeling regulations to limit FS consumption among the population of Saudi Arabia [17,18]. However, the problem of high consumption of FS remains despite efforts that have been made [3].

Limited knowledge of the FS recommendation is one important factor explaining the high consumption of FS [19,20]. For example, a female parent, a female child, and a higher education level were found to be linked to high recognition level of FS [19]. Understanding the link between maternal knowledge, attitude, and practices toward FS is crucial to design effective interventions aiming to reduce the consumption of FS among Saudi children.

During childhood, parents play a significant role in shaping the dietary behaviors of children. The impact of maternal feeding practices on children’s dietary intake has been previously established [21,22]. Maternal adherence to a healthy lifestyle, including a high-quality diet, and the utilization of healthy feeding practices have been shown to be effective in promoting positive dietary behaviors in children [23,24]. However, research addressing factors related to the consumption of FS among children in Saudi Arabia is lacking. Hence, the present study aimed to evaluate maternal knowledge, attitude, and practices related to FS and the associations with children’s intake of FS.

## 2. Materials and Methods

In this cross-sectional study, a minimum of 365 healthy children aged between 6–12 years and their mothers were needed based on an effect size (expected correlation coefficient between maternal knowledge and children intake of FS) of 0.20, 99.9 confidence level (two-sided test), and 90% power [25]. Exclusion criteria include children with allergies, chronic diseases, non-Saudi, sibling of a child who is already part of this study, and children who reside outside of Saudi Arabia within the past three months. Initially, we recruited 539 children and their mothers online through different social media channels (Instagram, Telegram, Twitter, WhatsApp, and Facebook). An invitation for participation was accompanied by a short online survey that collected demographic data [age of mother and child, child’s sex, maternal education level, and family income per month] and assessed maternal knowledge, attitude, and practices toward FS. A phone interview was scheduled with each mother within one week of collecting the initial data. The online survey and the phone interview were all administered in Arabic. The ethical approval for this study was granted from the ethical review board of the College of Applied Medical Sciences, Taibah University (certificate no. 2020/55/202/CLN). Informed consent was obtained online from all mothers involved in the study before collecting children’s data.

### 2.1. Assessment of Children’s Intake of Free Sugar

Data concerning children’s intake of FS were collected from mothers during the phone interviews. FS intake among children was assessed using a previously validated food frequency questionnaire (FFQ) that was specifically designed to assess the habitual intake of FS among children in Saudi Arabia [26]. The FFQ included 12 food groups and 40 food items. Frequency of consumption was recorded as follows: daily (6 times or more, 4–5 times, 2–3 times- once); weekly (5–6 times, 2–4 times, once); monthly (1–3 times, less than once). A template table presenting the response options (frequency of consumption) along with a guide to estimate portion sizes of food consumed was sent to mothers during the phone interview via WhatsApp/text message to improve the accuracy of responses to the FFQ. The questionnaire provided estimates concerning quantities of FS intake per day coming from liquid food sources, solid food sources, and total FS intake.

### 2.2. Assessment of Maternal Knowledge Related to Free Sugar

Maternal knowledge related to FS was assessed using 11 items as follows:

(1) Do you think eating too much FS is bad for your child’s health?

Response options were “Yes” and “No”. Mothers who responded “Yes” were awarded a score of one.

(2) Free sugar is:
  (2.1.) Sugar added to coffee and tea;
  (2.2.) Sugar added to food during processing or cooking;
  (2.3.) Sugar used to prepare sweets;
  (2.4.) Sugar exist in fruits and milk.
(3) The following food contains a large amount of free sugar:
  (3.1.) Diet Pepsi;
  (3.2.) Cookies;
  (3.3.) Milk;
  (3.4.) Fruit drinks;
  (3.5.) Toast bread and buns;
  (3.6.) Strawberry flavored Greek yogurt.

Mothers were instructed to select multiple responses as applicable for items included in sections two and three. Mothers who selected responses 2.1, 2.2, 2.3, 3.2, 3.4, and 3.6 were awarded a score of one for each question. A zero score per question was awarded to mothers who selected items 2.4, 3.1, 3.3, and 3.5, and a score of one was awarded for each of these items if the items were not selected. The total knowledge score was calculated (total score ranged between zero and 11), wherein a score of 11 indicated the highest level of knowledge on added sugar.

### 2.3. Assessment of Maternal Attitude to Limit Children’s Intake of Free Sugar

Maternal attitude towards limiting children’s FS intake was assessed using three items as follows:

(1) Are you trying to limit the purchase of foods that are high in free sugar?
(2) Are you trying to limit your child’s intake of foods that are high in free sugar?
(3) Are you trying to provide healthy food options for your child to replace foods that are high in free sugar?

Responses to all three items were “Yes” or “No”. Mothers who responded “Yes” were awarded a score of one per item, whereas a score of zero was awarded if mothers responded as “No” to any of the items. The total attitude score was later calculated (total scores ranged between zero and three).

### 2.4. Assessment of Maternal Practices to Limit Children’s Intake of Free Sugar

Maternal practices related to limiting children’s intake of FS were assessed using positive behaviors, and consisted of three items as follows:

(1) How often do you read the nutrition fact label of your child’s favorite products to determine the amount of free sugar intake?

Responses to this item were “Always” (awarded two scores); “Sometimes” (awarded one score); “Never” (recorded as a zero score).

(2) Are you discussing with your child the importance of replacing foods that are high in free sugar with healthy food options?

Responses to this item were “Yes” (awarded one score) and “No” (recorded as a zero score).

(3) Mother successfully limits/controls her child’s free sugar intake (assessed using the FFQ).

A score of one was awarded if the mother successfully limits/controls her child’s intake of FS to meet the recommendation of the World Health Organization (WHO) and the Ministry of Health in Saudi Arabia (MOH) (<25 g of FS per day) [27,28], whereas a score of zero was recorded if the mother did not limit/control her child’s intake of FS to meet the WHO/MOH recommendation (≥25 g of FS per day).

Scoring of maternal practices toward limiting children’s consumption of FS ranged between zero and three.

### 2.5. Statistical Analyses

Data for continuous variables included in this study are presented as median [interquartile range (IQR)] and mean ± standard deviation (SD), while data for categorical variables are presented as frequency and percentage (%). The Shapiro–Wilk test was used to assess the normality of all continuous variables. Data of maternal knowledge, attitude, and practices toward FS were skewed; thus, non-parametric tests were used to analyze data presented in this study. Spearman’s correlation test was used to assess the correlations between maternal knowledge, attitude, and practices toward FS. The Mann–Whitney and Kruskal Wallis tests were used to explore differences in mean scores of maternal knowledge, attitude, and practices toward FS within the categorical variables. Pairwise comparisons were performed to further investigate the significant associations found in the Kruskal Wallis test. Multiple linear regression analyses were performed to investigate if maternal knowledge, attitude, and practices toward FS predict children’s intake of FS after adjusting for children’s age and sex. Tests used in this study were two-tailed with a significance level of 95%. We corrected for multiple testing using the Bonferroni adjustment method when the significance of the pairwise comparisons were assessed. The SPSS (version 20, SPSS, Inc., Chicago, IL, USA) was used for data analysis.

## 3. Results

### 3.1. Characteristics of the Study Sample

Children with allergies and chronic diseases were excluded (*n* = 36, 6.68%). A total of 79 mothers (14.7%) were excluded due to missing dietary data (no phone interview was conducted) for one of the following reasons: (1) mother did not provide her contact information; (2) mother did not respond to text messages and reminders to schedule the phone interview to collect data concerning child’s FS intake. The final analysis of this study included data of 424 children and their mothers. Detailed data of the characteristics of children and their mothers are presented in Table 1.

### 3.2. Maternal Knowledge, Attitude, and Practices toward Free Sugar

Items included to assess maternal knowledge, attitude, and practices toward FS are provided in Table 2. The median of knowledge was 7.00 (6.00–8.00) out of 11.0. The median score of maternal attitude to limit children’s intake of FS was 2.00 (1.00–3.00) out of a total score of 3.00. The median score of maternal practices to limit children’s intake of FS was 2.00 (2.00–2.00) out of a total score of 4.00.

Spearman’s correlation test showed a negligible correlation between maternal knowledge and attitude toward FS (r_s_ = 0.20; *p* < 0.001) and between maternal knowledge and practices toward FS (r_s_ = 0.18; *p* < 0.001). A low positive correlation was observed between maternal attitude and practices toward limiting FS intake (r_s_ = 0.35; *p* < 0.001).

### 3.3. Maternal Knowledge, Attitude, and Practices toward Free Sugar and the Associations with Sociodemographic Characteristics

Detailed data concerning the associations between maternal knowledge, attitude, and practices related to FS and children’s characteristics are presented in Table 3. The mean maternal knowledge score towards FS was significantly different across the different regions in Saudi Arabia (*p* = 0.015). After using the Bonferroni adjustment method to correct for multiple testing, the pairwise comparisons showed a significantly higher mean of knowledge score among mothers in the Eastern region as compared to mothers in the Western region (7.83 ± 1.66 vs. 7.07 ± 1.55, respectively, *p* = 0.018). The mean maternal knowledge score toward FS was similar for all other sociodemographic variables. The mean maternal attitude score related to limiting FS intake was similar for all sociodemographic variables. The mean maternal practices score related to limiting FS intake was significantly different across regions in Saudi Arabia (*p* = 0.005). After using the Bonferroni adjustment method to correct for multiple testing, the pairwise comparisons showed significantly higher mean scores of practices among mothers in the Eastern region as compared to mothers in the Western region (2.17 ± 0.64 vs. 1.83 ± 0.73, respectively, *p* = 0.005). In addition, unemployed mothers had a higher mean maternal practices score toward limiting the consumption of FS compared to employed mothers (2.02 ± 0.64 vs. 1.77 ± 0.72, respectively, *p* < 0.001). The mean score of maternal practices related to limiting FS intake was similar for all other characteristics of children included in this study.

### 3.4. Maternal Knowledge, Attitude, and Practices Related to Free Sugar and the Associations with Children’s Intake of Free Sugar

Multiple linear regression analysis was performed to investigate the association between maternal knowledge, attitude, and practices related to FS and children’s intake of FS adjusting for children’s age and sex (Table 4). Maternal knowledge toward FS was not found to be associated with children’s intake of FS (from liquid food sources, solid food sources, and total FS from all food sources). Positive maternal attitude related to limiting FS intake predicted lower intake of FS from solid food sources in children (B: −5.73, SE: 2.07 [95% confidence interval (CI): −9.79 to −1.66], R-square = 0.02) and total FS from all food sources (B: −7.60, SE: 3.01 [95% CI: −13.5 to −1.68], R-square = 0.03), but no association with children’s intake of FS from liquid food sources was observed. Positive maternal practices related to limiting FS intake predicted lower intake of FS from solid food sources in children (B: −6.85, SE: 2.57 [95% CI: −11.9 to −1.80], R-square = 0.02) and total FS from all food sources (B: −7.92, SE: 3.74 [95% CI: −15.3 to −0.56], R-square = 0.02). Maternal practices related to FS were not associated with children’s intake of FS coming from liquid food sources.

## 4. Discussion

Limited knowledge about FS was observed among the mothers included in this study. Maternal knowledge was not correlated with maternal attitude or practices related to limiting the intake of FS. Maternal knowledge towards FS did not predict children’s intake of FS, whereas maternal attitude and practices to limit the consumption of FS predicted lower intake of FS among Saudi children, specifically FS coming from solid food sources.

The limited knowledge related to FS reported among mothers in our study has also been reported in other settings. A study conducted in China showed limited maternal knowledge related to FS [19]. In Ireland, a study also shows limited knowledge related to FS among adults where 65% of participants did not know the WHO recommendation for FS [20]. In fact, knowledge regarding FS might be influenced by many factors. The current food labeling practices in the Saudi market are inadequate [29], but notable progress has been observed [30,31]. For example, the Saudi government has implemented a policy for establishing front-of-pack nutrition labeling requirements and the FS nutritional labeling requirement [18,32,33]. Clear information provided in nutrition labels can help in increasing knowledge regarding FS content in prepackaged foods. However, improving knowledge of how to read nutrition labels is needed, as limited interpretation skills of nutrition labels have been reported previously in Saudi Arabia [34]. It is noteworthy that obtaining information from creditable sources is very important, especially with the wide use of social media applications, where inaccurate or even incorrect information is posted. Previous research in Ghana conducted among young adults reported that nutrition-related information is mostly obtained from online resources, whereas the least used source was healthcare professionals, e.g., nutritionists [35]. Thus, social media has been used effectively by national and international health organizations to deliver health-related messages to the public to raise awareness and improve knowledge.

In this study, the univariate analysis revealed that unemployed mothers have better practices related to limiting children’s intake of FS. This finding could be explained by the greater time availability for stay-at-home mothers. It has been noted that time constraints are the most common reasons for negative nutrition-related practices, e.g., not reading food labels [34]. Therefore, unemployed mothers who have more time on their hands can be more inclined to better nutrition-related practices, including reading food labels, discussing with children the importance of replacing less healthy foods with healthful food options, and limiting/controlling their children’s intake of FS.

Despite their limited knowledge of FS, mothers were making efforts to limit children’s intake of FS. Yet, the majority of children exceeded the recommendation of the WHO/MOH. The excessive consumption of FS could be due to the affordability, palatability, and convenience of sugary foods [13,14,16]. In fact, it is evident that the sweet taste of food is preferred, particularly among children [11,12]. Our data also showed that maternal attitude and practices related to limiting FS only predicted children’s intake of FS from solid food sources and total FS, but not FS coming from liquid food sources. The lack of association between maternal attitude and practices to limit children’s intake of FS from liquid food sources could be due to efforts that have been made by the Saudi government to limit the intake of sugary drinks [17]. In fact, the sugary drink tax policy implemented in 2017 might result in limiting the consumption of FS from liquid food sources. Liquid food sources of FS were found to be the greatest contributor to total energy intake among many populations [1,36]. However, data regarding top food sources of FS among children in Saudi Arabia are lacking. Previous research showed high consumption of sweet snacks and candies among children and university students [37,38].

Maternal attitude and practices toward FS can influence children’s intake of FS despite their level of knowledge. Efforts made by mothers to limit children’s intake of FS can help in eliminating many food sources that contain high quantities of FS and replace them with healthy food options. Previous nutrition education interventions conducted among preschoolers and their parents were found to be effective in reducing children’s consumption of FS and increasing the density of important nutrients, including protein, fiber, potassium, iron, and zinc [39]. However, replacing sugary foods with healthy food options, e.g., fruits and vegetables, might be more costly [15,16].

This is the first study in the region to explore maternal knowledge, attitude, and practices related to FS in relation to children’s intake of FS. The FS intake was assessed using a validated FFQ that can estimate the habitual intake of FS among Saudi children. The study is limited by the convenience sampling method used. Thus, the findings of this study might be generalizable only to mothers who commonly use social media channels.

## 5. Conclusions

Despite the limited knowledge observed pertaining to FS among mothers in Saudi Arabia, these mothers were making efforts to limit their children’s consumption of FS. Future research should be directed towards exploring other potential predictors of FS intake among Saudi children. In addition, longitudinal associations between knowledge, attitude, and practices related to FS intake should be further explored among individuals from different age groups in Saudi Arabia. Nutrition education interventions to improve maternal knowledge on FS and its food sources are urgently needed.

## Figures and Tables

**Table 1:** Characteristics of children and their mothers (*n* = 424).

Variable	*n*	%
Region of residency		
Western region	242	57.1
Central region	56	13.2
Eastern region	53	12.5
Other regions	73	17.2
Age		
6–7 years	144	34.0
8–9 years	126	29.7
10–12 years	154	36.3
Sex		
Boys	210	49.5
Girls	214	50.5
Order of child		
Older child	139	32.8
Middle child	135	31.8
Younger child	128	30.2
Only child	22	5.20
Maternal age		
≤30 years	76	17.9
31–40 years	239	56.4
>40 years	109	25.7
Maternal education level		
≤High school	105	24.8
University degree	270	63.7
Postgraduate degree	49	11.6
Maternal employment status		
Employed	173	40.8
Unemployed	251	59.2
Family income per month in SR ^1^		
<4000	29	6.80
4000–10,000	166	39.2
>10,000	229	54.0

^1^ SR: Saudi Riyal ($1 = SR 3.75).

**Table 2:** Maternal knowledge, attitude, and practices toward free sugar (*n* = 424).

	Item	*n*	%
	Maternal knowledge toward free sugar		
1	Do you think eating too much free sugar is bad for your child’s health?		
	Yes ^1^	402	94.8
	No	22	5.20
2	What is free sugar?		
2.1	Sugar added to coffee and tea		
	Yes ^1^	147	34.7
	No	277	65.3
2.2	Sugar added to food during processing or cooking		
	Yes ^1^	302	71.2
	No	122	28.8
2.3	Sugar used to prepare sweets		
	Yes ^1^	194	45.8
	No	230	54.2
2.4	Sugar exist in fruits and milk		
	Yes	29	6.80
	No ^1^	395	93.2
3	The following food contains a large amount of free sugar:		
3.1	Diet Pepsi		
	Yes	254	59.9
	No ^1^	170	40.1
3.2	Cookies		
	Yes ^1^	344	81.1
	No	80	18.9
3.3	Plain milk		
	Yes	16	3.80
	No ^1^	408	96.2
3.4	Fruit drinks		
	Yes ^1^	367	86.6
	No	57	13.4
3.5	Toast bread and buns		
	Yes	172	40.6
	No ^1^	252	59.4
3.6	Strawberry flavored Greek yogurt		
	Yes ^1^	78	18.4
	No	346	81.6
	Maternal attitude to limit children’s intake of free sugar		
1	Are you trying to limit the purchase of foods that are high in free sugar?		
	Yes ^1^	174	41.0
	No	250	59.0
2	Are you trying to limit your child’s intake of foods high in free sugar?		
	Yes ^1^	289	68.2
	No	135	31.8
3	Are you trying to provide healthy food options for your child to replace foods high in free sugar?		
	Yes ^1^	394	92.9
	No	30	7.10
	Maternal practices to limit children’s intake of free sugar		
1	How often do you read the nutrition fact label of your child’s favorite products to determine the amount of free sugar intake?		
	Always	64	15.1
	Sometimes	283	66.7
	Never	77	18.2
2	Are you discussing with your child the importance of replacing foods high in free sugar with healthy food options?		
	Yes ^1^	396	93.4
	No	28	6.60
3	Mother successfully limit/control her child free sugar intake		
	Child’s intake of free sugar < 25 g per day ^1^	7	1.70
	Child’s intake of free sugar ≥ 25 g per day	417	98.3

^1^ Response awarded a score of one.

**Table 3:** Associations between maternal knowledge, attitude, and practices related to free sugar and children’s characteristics (*n* = 424).

Characteristics	Maternal Knowledge Related to Free Sugar ^1^ Score out of 11	Maternal Attitude to Limit Free Sugar Intake ^1^ Score out of 3	Maternal Practices to Limit Free Sugar Intake ^1^ Score out of 4
Region of residency			
Western region	7.07 ± 1.55 7.00 [6.00–8.00]	1.98 ± 0.89 2.00 [1.00–3.00]	1.83 ± 0.73 2.00 [1.00–2.00]
Central region	7.43 ± 1.35 7.50 [7.00–8.00]	2.07 ± 0.81 2.00 [1.25–3.00]	1.91 ± 0.61 2.00 [2.00–2.00]
Eastern region	7.83 ± 1.66 8.00 [7.00–9.00]	1.98 ± 0.75 2.00 [2.00–2.00]	2.17 ± 0.64 2.00 [2.00–3.00]
Other regions	7.11 ± 1.48 7.00 [6.00–8.00]	2.16 ± 0.80 2.00 [2.00–3.00]	2.03 ± 0.55 2.00 [2.00–2.00]
*p*-value	0.015 ^3^	0.405	0.005 ^3^
Age			
6–7 years	7.19 ± 1.56 7.00 [6.00–8.00]	2.00 ± 0.78 2.00 [2.00–3.00]	1.92 ± 0.67 2.00 [2.00–2.00]
8–9 years	7.42 ± 1.54 7.00 [7.00–8.00]	2.04 ± 0.88 2.00 [1.00–3.00]	1.94 ± 0.70 2.00 [2.00–2.00]
10–12 years	7.09 ± 1.52 7.00 [6.00–8.00]	2.03 ± 0.89 2.00 [1.00–3.00]	1.90 ± 0.69 2.00 [2.00–2.00]
*p*-value	0.497	0.122	0.114
Sex			
Boys	7.09 ± 1.57 7.00 [6.00–8.00]	2.01 ± 0.87 2.00 [1.00–3.00]	1.89 ± 0.66 2.00 [2.00–2.00]
Girls	7.35 ± 1.51 7.00 [6.00–8.00]	2.03 ± 0.82 2.00 [2.00–3.00]	1.95 ± 0.71 2.00 [2.00–2.00]
*p*-value	0.077	0.876	0.347
Order of child			
Older child	7.15 ± 1.56 7.00 [6.00–8.00]	2.12 ± 0.80 2.00 [2.00–3.00]	1.94 ± 0.66 2.00 [2.00–2.00]
Middle child	7.27 ± 1.50 7.00 [6.00–8.00]	2.09 ± 0.85 2.00 [2.00–3.00]	1.99 ± 0.71 2.00 [2.00–2.00]
Younger child	7.13 ± 1.50 7.00 [6.00–8.00]	1.87 ± 0.89 2.00 [1.00–3.00]	1.84 ± 0.70 2.00 [2.00–2.00]
Only child	7.86 ± 1.81 8.00 [6.00–9.00]	1.91 ± 0.75 2.00 [1.00–2.25]	1.86 ± 0.56 2.00 [2.00–2.00]
*p*-value	0.236	0.074	0.322
Maternal age			
≤30 years	7.41 ± 1.38 7.00 [7.00–8.00]	2.01 ± 0.77 2.00 [2.00–3.00]	1.96 ± 0.74 2.00 [2.00–2.00]
31–40 years	7.17 ± 1.60 7.00 [6.00–8.00]	2.09 ± 0.84 2.00 [2.00–3.00]	1.97 ± 0.65 2.00 [2.00–2.00]
>40 years	7.19 ± 1.52 7.00 [6.00–8.00]	1.88 ± 0.90 2.00 [1.00–3.00]	1.79 ± 0.71 2.00 [1.00–2.00]
*p*-value	0.497	0.122	0.114
Maternal education level			
≤High school	7.18 ± 1.49 7.00 [6.00–8.00]	1.98 ± 0.75 02.00 [2.00–2.−0]	1.98 ± 0.59 2.00 [2.00–2.00]
University	7.24 ± 1.49 7.00 [6.00–8.00]	2.00 ± 0.86 2.00 [1.00–3.00]	1.90 ± 0.70 2.00 [2.00–2.00]
Postgraduate	3.16 ± 1.94 7.00 [5.50–9.00]	2.20 ± 0.93 2.00 [2.00–3.00]	1.92 ± 0.79 2.00 [2.00–2.00]
*p*-value	0.985	0.124	0.565
Maternal employment ststus			
Employed	3.18 ± 1.62 7.00 [6.00–9.00]	1.98 ± 0.91 2.00 [1.00–3.00]	1.77 ± 0.72 2.00 [1.00–2.00]
Unemployed	7.25 ± 1.49 7.00 [6.00–8.00]	2.05 ± 0.80 2.00 [2.00–3.00]	2.02 ± 0.64 2.00 [2.00–2.00]
*p*-value	0.625	0.528	<0.001 ^3^
Family income per month in SR ^2^			
<4000	7.00 ± 1.79 7.00 [6.00–8.00]	1.83 ± 0.85 2.00 [1.00–2.00]	1.97 ± 0.73 2.00 [2.00–2.00]
4000–10,000	7.39 ± 1.45 7.00 [6.00–8.00]	2.05 ± 0.81 2.00 [2.00–3.00]	1.95 ± 0.67 2.00 [2.00–2.00]
>10,000	7.12 ± 1.57 7.00 [6.00–8.00]	2.02 ± 0.87 2.00 [1.00–3.00]	1.90 ± 0.69 2.00 [2.00–2.00]
*p*-value	0.293	0.428	0.813

^1^ The numbers presented are mean ± SD and median [IQR]. ^2^ SR: Saudi Riyal [$1 = SR 3.75]. ^3^ *p*-value was considered statistically significant at the 95% confidence level.

**Table 4:** Multiple linear regression analysis of associations between maternal knowledge, attitude, and practices related to free sugar and children’s intake of free sugar ^1^.

	Beta Estimate	Standard Error	95% Confidence Interval	*p*-Value	R-Square
Maternal knowledge related to free sugar					
Free sugar intake from liquid food sources, g/day	−0.72	0.89	−2.47 to 1.03	0.418	0.02
Free sugar intake from solid food sources, g/day	−0.70	1.16	−2.97 to 1.57	0.543	0.00
Total free sugar intake, g/day	−1.53	1.68	−4.83 to 1.76	0.361	0.01
Maternal attitude to limit children’s intake of free sugar					
Free sugar intake from liquid food sources, g/day	−2.03	1.60	−5.18 to 1.12	0.206	0.02
Free sugar intake from solid food sources, g/day	−5.73	2.07	−9.79 to −1.66	0.006 ^2^	0.02
Total free sugar intake, g/day	−7.60	3.01	−13.5 to −1.68	0.012 ^2^	0.03
Maternal practices to limit children’s intake of free sugar					
Free sugar intake from liquid food sources, g/day	−0.97	1.99	−4.89 to 2.95	0.627	0.02
Free sugar intake from solid food sources, g/day	−6.85	2.57	−11.9 to −1.80	0.008 ^2^	0.02
Total free sugar intake, g/day	−7.92	3.74	−15.3 to −0.56	0.035 ^2^	0.02

^1^ All models were adjusted for children’s age and sex. ^2^ *p*-value was considered statistically significant at the 95% confidence level.

## Data Availability

The datasets used and/or analyzed during the current study are available from the corresponding author on reasonable request.

## References

[1] Bailey R.L., Fulgoni V.L., Cowan A.E., Gaine P.C. (2018). Sources of Added Sugars in Young Children, Adolescents, and Adults with Low and High Intakes of Added Sugars. Nutrients.

[2] Jomaa L., Hamamji S., Kharroubi S., Diab-El-Harakeh M., Al Zahraa Chokor F., Nasreddine L. (2021). Dietary Intakes, Sources, and Determinants of Free Sugars amongst Lebanese Children and Adolescents: Findings from Two National Surveys. Eur. J. Nutr..

[3] Mumena W. (2021). Consumption of Free Sugar Predicts Nutrient Intake of Saudi Children. Front Nutr..

[4] Rippe J.M., Angelopoulos T.J. (2016). Relationship between Added Sugars Consumption and Chronic Disease Risk Factors: Current Understanding. Nutrients.

[5] Vos M.B., Kaar J.L., Welsh J.A., Van Horn L.V., Feig D.I., Anderson C.A.M., Patel M.J., Cruz Munos J., Krebs N.F., Xanthakos S.A. (2017). Added Sugars and Cardiovascular Disease Risk in Children: A Scientific Statement from the American Heart Association. Circulation.

[6] Kaartinen N.E., Simila M.E., Kanerva N., Valsta L.M., Harald K., Männisto S. (2017). Naturally Occurring and Added Sugar in Relation to Macronutrient Intake and Food Consumption: Results from a Population-Based Study in Adults. J. Nutr. Sci..

[7] Gibson S., Boyd A. (2009). Associations between Added Sugars and Micronutrient Intakes and Status: Further Analysis of Data from the National Diet and Nutrition Survey of Young People Aged 4 to 18 Years. Br. J. Nutr..

[8] Louie J.C.Y., Tapsell L.C. (2015). Association between Intake of Total vs. Added Sugar on Diet Quality: A Systematic Review. Nutr. Rev..

[9] Rivera J.A., Hotz C., González-Cossío T., Neufeld L., García-Guerra A. (2003). The Effect of Micronutrient Deficiencies on Child Growth: A Review of Results from Community-Based Supplementation Trials. J. Nutr..

[10] Rosenstock I.M. (1974). Historical Origins of the Health Belief Model. Heal. Educ. Behav..

[11] Hoffman A.C., Salgado R.V., Dresler C., Faller R.W., Bartlett C. (2016). Flavour Preferences in Youth versus Adults: A Review. Tob. Control.

[12] Ventura A.K., Mennella J.A. (2011). Innate and Learned Preferences for Sweet Taste during Childhood. Curr. Opin. Clin. Nutr. Metab. Care.

[13] Yamamoto T. (2003). Brain Mechanisms of Sweetness and Palatability of Sugars. Nutr. Rev..

[14] United States Department of Agriculture (2018). Consumers Balance Time and Money in Purchasing Convenience Foods. https://www.ers.usda.gov/webdocs/publications/89344/err251_summary.pdf?v=261.8.

[15] Darmon N., Drewnowski A. (2015). Contribution of Food Prices and Diet Cost to Socioeconomic Disparities in Diet Quality and Health: A Systematic Review and Analysis. Nutr. Rev..

[16] Drewnowski A. (2003). Fat and Sugar: An Economic Analysis. J. Nutr..

[17] Alsukait R., Wilde P., Bleich S., Singh G., Folta S. (2019). Impact of Saudi Arabia’s Sugary Drink Tax on Prices and Purchases (P10-066-19). Curr. Dev. Nutr..

[18] Saudi Food and Drug Authority (2018). SFDA Healthy Food Strategy.

[19] Tang Q., Lin Q., Yang Q., Sun M., Liu H., Yang L. (2020). Knowledge, Attitude, and Practice of Adolescent Parents on Free Sugar and Influencing Factors about Recognition. Int. J. Environ. Res. Public Health.

[20] Tierney M., Gallagher A., Giotis E., Pentieva K. (2017). An Online Survey on Consumer Knowledge and Understanding of Added Sugars. Nutrients.

[21] Savage J.S., Fisher J.O., Birch L.L. (2007). Parental Influence on Eating Behavior: Conception to Adolescence. J. Law Med. Ethics.

[22] Daniels L.A. (2019). Feeding Practices and Parenting: A Pathway to Child Health and Family Happiness. Ann. Nutr. Metab..

[23] Dhana K., Haines J., Liu G., Zhang C., Wang X., Field A.E., Chavarro J.E., Sun Q. (2018). Association between Maternal Adherence to Healthy Lifestyle Practices and Risk of Obesity in Offspring: Results from Two Prospective Cohort Studies of Mother-Child Pairs in the United States. BMJ.

[24] Mahmood L., Flores-Barrantes P., Moreno L.A., Manios Y., Gonzalez-Gil E.M. (2021). The Influence of Parental Dietary Behaviors and Practices on Children’s Eating Habits. Nutrients.

[25] Hulley S., Cummings S., Browner W. (2015). Designing Clinical Research.

[26] Mumena W.A., Kutbi H.A. Development of a Food Frequency Questionnaire for Assessing Habitual Intake of Free Sugar among Children in Saudi Arabia.

[27] World Health Organization (2015). WHO Calls on Countries to Reduce Sugars Intake among Adults and Children. https://www.who.int/mediacentre/news/releases/2015/sugar-guideline/en/.

[28] Ministry of Health Saudi Arabia Healthy Food Guidelines for Health Practitioners. https://www.moh.gov.sa/Ministry/About/HealthPolicies/Healthy-Food-Guidelines-for-Health-Practitioners.pdf.

[29] AlMughthem A., Jradi H., Bawazir A. (2020). Nutrition Food Labeling in the Saudi Market between Compliance and Relaxing Policy. Asian J. Med. Health.

[30] Saudi Food and Drug Authority-Food Sector (2011). Rejected Claims on Labeling of Foodstuff. https://old.sfda.gov.sa/en/food/circulations/Circulations/SFDA_Announcement_Foodstuff.pdf.

[31] Saudi Food and Drug Authority Food Regulations. https://sfda.gov.sa/en/regulations?keys=&regulation_type=All&date%5Bmin%5D=&date%5Bmax%5D=&tags=1.

[32] Al-Jawaldeh A., Rayner M., Julia C., Elmadfa I., Hammerich A., McColl K. (2020). Improving Nutrition Information in the Eastern Mediterranean Region: Implementation of Front-of-Pack Nutrition Labelling. Nutrients.

[33] United States Department of Agriculture (2019). Saudi Arabia- Food and Agricultural Import Regulations and Standards Report: FAIRS Annual Country Report.

[34] Al-Barqi R., Al-Salem Y., Mahrous L., Abu Abat E., Al-Quraishi R., Benajiba N. (2020). Understanding Barriers towards the Use of Food Labels among Saudi Female College Students. Malays. J. Nutr..

[35] Quaidoo E.Y., Ohemeng A., Amankwah-Poku M. (2018). Sources of Nutrition Information and Level of Nutrition Knowledge among Young Adults in the Accra Metropolis. BMC Public Health.

[36] Lei L., Rangan A., Flood V.M., Louie J.C.Y. (2016). Dietary Intake and Food Sources of Added Sugar in the Australian Population. Br. J. Nutr..

[37] Mumena W.A., Alamri A.A., Mahrous A.A., Alharbi B.M., Almohaimeed J.S., Hakeem M.I., Kutbi H.A. (2020). Knowledge, Attitudes, and Practices toward Added Sugar Consumption among Female Undergraduate Students in Madinah, Saudi Arabia: A Cross-Sectional Study. Nutrition.

[38] Alsubaie A.S.R. (2017). Consumption and Correlates of Sweet Foods, Carbonated Beverages, and Energy Drinks among Primary School Children in Saudi Arabia. Saudi Med. J..

[39] Yeom M.Y., Cho Y.O. (2019). Nutrition Education Discouraging Sugar Intake Results in Higher Nutrient Density in Diets of Pre-School Children. Nutr. Res. Pract..

